# Experimental support for reclassification of the light scattering second virial coefficient from macromolecular solutions as a hydrodynamic parameter

**DOI:** 10.1007/s00249-023-01665-w

**Published:** 2023-07-18

**Authors:** Donald J. Winzor, Vlad Dinu, David J. Scott, Stephen E. Harding

**Affiliations:** 1 School of Chemistry and Molecular Biosciences, University of Queensland, Brisbane, QLD 4072 Australia; 2 National Centre for Macromolecular Hydrodynamics, School of Biosciences, University of Nottingham, Sutton Bonington, LE12 5RD UK; 3 Research Complex at Harwell, Rutherford Appleton Laboratory, OX11 0FA UK

**Keywords:** Dynamic light scattering, Hydrodynamics, Lysozyme, Monoclonal IgG antibodies, Second virial coefficient, Statistical mechanics, Static light scattering, Thermodynamic nonideality

## Abstract

This investigation examines the source of the disparity between experimental values of the light scattering second virial coefficient ${A}_{2}$ (mL.mol/g^2^) for proteins and those predicted on the statistical mechanical basis of excluded volume. A much better theoretical description of published results for lysozyme is obtained by considering the experimental parameters to monitor the difference between the thermodynamic excluded volume term and its hydrodynamic counterpart. This involves a combination of parameters quantifying concentration dependence of the translational diffusion coefficient obtained from dynamic light scattering measurements. That finding is shown to account for observations of a strong correlation between ${A}_{2}{M}_{2}$ (mL/g), where *M*_2_ is the molar mass (molecular weight) of the macromolecule and the diffusion concentration parameter ${k}_{D}$ (mL/g). On the grounds that ${k}_{D}$ is regarded as a hydrodynamic parameter, the same status should be accorded the light scattering second virial coefficient rather than its current incorrect thermodynamic designation as ${B}_{2}$ (mL.mol/g^2^), or just *B*, the osmotic second virial coefficient for protein self-interaction.

## Introduction

In accordance with standard textbook doctrine (Tanford 1961; Cantor and Schimmel 1980; Van Holde 1985) the nonideality parameter ${A}_{2}$ (mL.mol.g^−2^) determined from the linear concentration dependence of “classical” or “total intensity” light scattering intensity measurements for protein solutions has routinely been identified with ${B}_{2},$ the osmotic second virial coefficient (mL mol ${\mathrm{g}}^{-2}$) for solute self-interaction. However, that designation was called into question (Deszczynski et al. 2006; Winzor et al. 2007) as the result of reports of negative values (George and Wilson 1994; Muschol and Rosenberger 1995; Rosenbaum and Zukoski 1996)—a finding incompatible with the interpretation of thermodynamic nonideality on the statistical-mechanical basis of excluded volume (McMillan and Mayer 1945; Hill 1968). Experimental support for that contention has come from rigorous estimation of the osmotic second virial coefficient for protein self-interaction by sedimentation equilibrium (Wills and Winzor 1992; Wills et al. 1993), which has yielded positive values of ${B}_{2}$ for lysozyme (Behlke and Ristau 1999), equine serum albumin (Desczynski et al. 2006) and ovalbumin (Winzor et al. 2007) under comparable conditions to those yielding negative ${A}_{2}$ values from “static” light scattering measurements (Muschol and Rosenberger 1995; Guo et al. 1999; Winzor et al. 2007). The thermodynamic second virial coefficient *B*_2_ when measured correctly from osmotic pressure or sedimentation equilibrium in the analytical ultracentrifuge (Hall et al 1999) is useful in the description of the thermodynamic nonideality behaviour of macromolecular solutions and in the elucidation of macromolecular shape, conformation in solution and solvation (Rallison and Harding 1985; Harding 1985; Winzor et al 2001; Serdyuk et al 2007 and references cited therein).

On the grounds that the quantitative expressions derived from a more rigorous thermodynamic treatment of the concentration dependence of static light scattering experiments still failed to generate the negative ${A}_{2 }$ versus *c*_2_ dependence (Winzor et al. 2007), we now explore the possibility that the linear concentration coefficient obtained from light scattering intensity measurements should be reclassified as a hydrodynamic steady-state parameter (Winzor et al. 2007).

## Theoretical considerations

Caution is required when defining the second virial coefficient for a macromolecular solution because of the different conditions under which the solute concentration can be varied (Winzor and Wills 1994). Theory has traditionally been written for the condition of osmotic equilibrium, which is a convenient standard to which changes in thermodynamic quantities can be referred. Choice of this standard condition then allows the establishment of a formal equivalence between the theory of imperfect gases (van der Waals 1873) and nonideal macromolecular solutions (Hill 1959; Wills and Winzor 2005).

## Macromolecular solutions: thermodynamic considerations

The inclusion of a single protein solute (species 2) in solvent (species 1) at constant temperature gives rise to one of two situations. In classical osmometry, for example, the chemical potential of solvent ($\mu_{1}$ ) in the protein-containing phase (α) and solvent phase (β) remains equal to that of solvent at atmospheric pressure; and hence complies with the condition of osmotic equilibrium discussed above. Because buffer components and additional small cosolutes are also in partition equilibrium between the two phases, they too can be regarded as part of the solvent, whereupon the solution becomes a single-solute system. A totally different situation arises in situations (as in light scattering measurements) where constant pressure *P* is the second constraint. Because the osmotic equilibrium condition is not met: the solvent chemical potential then becomes dependent upon the protein concentration. Consequently, separate definitions of the solute chemical potential ($\mu_{2}$) are required for the two situations (Hill 1959).

Under the constraints of constant temperature and solvent chemical potential the thermodynamic activity of the protein ${(z}_{2})$ is related to its molar concentration (${C}_{2}$) by the relationship (Hill 1959; Wills et al. 2015)

1 $$(\mu_{2} )_{{T,\mu_{1} }} = (\mu_{2}^{o} )_{{T,\mu_{1} }} + {\text{R}}T\ln z_{2} = (\mu_{2}^{o} )_{{T,\mu_{1} }} + {\text{R}}T\ln_{{\gamma_{2} }} C_{2}$$

where the solute thermodynamic activity z_2_ is a molar quantity in the sense that it equals the molar concentration ${C}_{2}$ in the ideal limit of infinite dilution (a situation denoted by superscript o); and is therefore most appropriately expressed as the product of ${C}_{2}$ and a dimensionless activity coefficient $\gamma_{2}$ . The osmotic pressure can be written in virial form as

2a $$\frac{\Pi}{{{\text{R}}T}}{\mkern 1mu} = {\mkern 1mu} {\mkern 1mu} C_{2} + B_{2} .M_{2}^{2} {\mkern 1mu} C^{2} + \ldots$$

where *M*_2_ is the molar mass of the solute (g.mol^−1^) and *B*_2_ is the second virial coefficient (mL.mol.g^−2^). Rewriting with the second virial coefficient *B*_2_ expressed in the dimensions of exclusion volume (mL.mol.^−1^) $B_{22} = B_{2 }.M_{2}^{2}$

2b $$\frac{\Pi}{{{\text{R}}T}} = \,C_{2} + B_{22 }.C_{2}^{2} + \ldots$$

These and other terms are summarised in Table 1.

**Table 1:** Symbols and abbreviations used, and equations in which they appear

Symbol	Meaning	Units	Equations
*M, M*_2_	molar mass of solute	g.mol^−1^	11,12,15,19,20
*A*_2_	2^nd^ light scattering virial coefficient	mL.mol.g^−2^	19
*B*_2_	2^nd^ thermodynamic virial coefficient	mL.mol.g^−2^	2,19
*B*_22_	*B*_2_ expressed as an exclusion volume = *B*_2_*M*_2_^2^	mL.mol^−1^	2,3,4,7,12
*k*_d_	concentration dependence of translational diffusion coefficient	mL.g^−1^	16,20
*C*_2_	molar concentration of solute	mol.mL^−1^	2,3
*c*_2_	weight (mass) concentration of solute	g.mL^−1^	20
*m*_2_	molal concentration of solute	mol.g^−1^	9,10,13
$\mu_{1} ,\,\,\mu_{2}$	solvent, solute chemical potential	erg.mol^−1^	1
$\mu_{1}^{ \circ },\mu_{2}^{ \circ }$	$\mu_{1} ,\,\mu_{2}$ value at infinite dilution	erg.mol^−1^	1
$\alpha ,\beta$	protein containing phase, solvent containing phase	–	
*z*_2_	thermodynamic activity of solute (molar scale)	mol.mL^−1^	1
*a*_2_	thermodynamic activity of solute (molal scale)	mol.g^−1^	8
$\gamma_{2} ,\,y_{2}$	dimensionless activity coefficient	–	1,8,10
Π	osmotic pressure	dyn.cm^−2^	
R	gas constant	8.314 × 10^7^ erg mol^−1^ K^−1^	1,6
*T*	absolute temperature	K	1,4,6
*R*	centre-to-centre distance	cm	4
*u*_22_	potential-of-mean-force between 2 molecules whose centres separated by *r*		4,6
k_B_	Boltzmann constant	1.3807 × 10^–16^ cm^2^.g.s^−2^ K^−1^	4
$N_{A}$	Avogadro’s number	6.0221 × 10^23^ mol^−1^	4,5,7,15,22
*Z*_2_	net valency	–	5
*R*_2_	spherical solute radius	cm	4,5,7
κ	Debye-Hückel inverse screening length	cm^−1^	5,7
*I*_m_	ionic strength (molar)	mol.L^−1^	5,7
*C*_22_, *C*_23_	molal 2^nd^ virial coefficient for protein–protein interaction, for protein-cosolute interaction	–	9.10
*m*_2_, *m*_3_	molal concentrations of solute, cosolute	mol.g^−1^	9,10
${\overline{v} }_{2}$	partial specific volume of solute	mL.g^−1^	15
$\rho_{1} ,\,\rho_{2}$	density of solvent, solute	g.mL^−1^	11,12
*D*, *D*_o_	translational diffusion coefficient, and *D* value at infinite dilution	cm^2^.s^−1^	17
ϕ	volume fraction of solute	–	14
$\lambda_{{\text{T}}} ,\,\lambda_{{\text{H}}}$	volume fraction dependence thermodynamic and hydrodynamic coefficients	–	14,16
HS	hard-sphere contribution	–	16
*v*_s_	swollen (hydrated) specific volume of solute	mL.g^−1^	16
$\lambda_{b}$	Oseen coefficient	–	15
$\eta ,\eta_{b}$	viscosity of solution, solvent	cm^−1^⋅g⋅s^−1^	17,18
$\left[ \eta \right]$	intrinsic viscosity of a macromolecule/polymer in solution	mL.g^−1^	18

Using a purely thermodynamic argument, Hill (1959) has shown that the expression

3 $$\ln \gamma_{2} = \,\,2B_{22}. C_{2} + \ldots$$

defines the molar activity coefficient. For a spherical solute with radius $R_{2}$ his second virial coefficient can be expressed in terms of the potential-of-mean-force, $u_{22}$, between two molecules separated by centre-to-centre distance *r* via the equations (McMillan and Mayer 1945; Mayer 1950; Hill 1968)

4a $$B_{22} = 2\pi {\text{N}}_{{\text{A}}} \left[ {\int_{0}^{{2R_{2} }} {r^{2} \,dr + } \int_{{2R_{2} }}^{\infty } {f_{22} \left( r \right)r^{2} dr} } \right]$$

4b $$f_{22} \left( r \right) = \exp \left[ { - u_{22} \left( r \right)/({\text{k}}_{{\text{B}}} T)} \right] - 1$$

where ${\mathrm{k}}_{\mathrm{B}}$ is the Boltzmann constant; and where Avogadro’s number $\left( {N_{A} } \right)$ converts the virial coefficient from a molecular to a molar basis. The first integral in Eq. (4a) accounts for the excluded volume for two uncharged spheres (the hard-sphere contribution $B_{22}^{HS}$), whereas the second integral accommodates the additional contribution of the perturbation of the chemical potential arising from electrostatic interaction $\left( {B_{22}^{EL} } \right)$ via the function $u_{22} \left( r \right)$ written in the form

5 $$\frac{{u_{22} \left( r \right)}}{{{\text{k}}_{{\text{B}}} T}}\,\, = \,\,\,\frac{{1000Z_{2}^{2} \,\kappa^{2} {\text{exp}}\,\,\left[ { - \kappa \left( {r - 2R_{2} } \right)} \right]}}{{8\pi {\text{N}}_{{\text{A}}} I_{M} \,(1 + \kappa R_{2} )^{2} r}}\,\,\,r \ge 2R_{2}$$

for a protein bearing net charge ${Z}_{2}$ (not to be confused with the activity *z*_2_). The factor of 1000 reflects calculation of the Debye–Hückel inverse screening length κ (in cm^−1^) as 3.27 × ${10}^{7}\sqrt{{I}_{M}}$, where ${I}_{M}$ is the ionic strength recorded on the conventional molar scale (mol.L^−1^). Solution of Eq. (4) by approximating the Mayer function as

6 $$f_{22} \left( r \right) = - u_{22} \left( r \right)/\left( {{\text{k}}_{{\text{B}}} T} \right) + [u_{22} \left( r \right)/\left( {{\text{k}}_{{\text{B}}} T} \right)]^{2} + \, \ldots$$

leads to the expression (Wills and Winzor 2009)

7 $$B_{22} = \,\,\,\frac{{16\pi {\text{N}}_{{\text{A}}} R_{2}^{3} }}{3}\, + \,\frac{{Z_{2}^{2} \left( {1 + 2\kappa R_{2} } \right)}}{{4I_{M} (1 + \kappa R_{2} )^{2} }} - \frac{{Z_{2}^{4} \left( {1000\kappa^{3} } \right)}}{{128I_{M}^{2} (1 + \kappa R_{2} )^{4} }} \ldots$$

which establishes that the osmotic second virial coefficient for macromolecule self-interaction can only assume positive values because of convergence of the series of charge-dependent terms with alternating sign.

For solutions with temperature and pressure as fixed constraints, the thermodynamic activity of the macromolecule (${a}_{2}$) is defined (Hill 1968) by the equation

8 $$(\mu_{2} )_{T,P} = \,(\mu_{2}^{o} )_{T,P} + {\text{R}}T\ln a_{2} = {\text{R}}T\,\, {\text{ln}}\,\,\left( {y_{2} m_{2} } \right)$$

in which ${a}_{2}$ is a molal quantity and therefore most appropriately expressed as the product of molal concentration ${m}_{2}$ and a dimensionless activity coefficient ${y}_{2}$. Under these conditions the counterparts of Eqs. (2) and (3) for the change in solvent chemical potential due to the addition of solvent become (Hill 1968; Wills et al 1993)

9 $$\frac{{(\mu_{1} )_{T,P} - (\mu_{1}^{o} )_{T,P} }}{{{\text{R}}T}}\,\,\, = \,\,m_{2} + C_{22}.m_{2}^{2} + \ldots$$

10 $$\ln y_{2} = 2C_{22}.m_{2} + \ldots$$

where ${C}_{22}$ is the molal second virial coefficient for macromolecule self-interaction. Unlike its molar counterpart ${B}_{22}$, the molal second virial coefficient (${C}_{22}$) is not generally amenable to statistical-mechanical interpretation. However, the assumption of solution incompressibility (an acceptable approximation for aqueous systems) allows the expression of a molal concentration in terms of its molar counterpart as

11 $$m_{2} \, = \,\frac{{C_{2} }}{{\rho_{1} \left( {1 - M_{2}. \overline{v}_{2} .C_{2} } \right)}}\, \approx \,\frac{{C_{2} }}{{\rho_{1} }}\left( {1 + M_{2}. \,\overline{v}_{2} \,.C_{2} + \ldots } \right)$$

where ${M}_{2}$ and ${\overline{v} }_{2}$ denote the molar mass and partial specific volume, respectively, of the protein, and where $\gamma_{1}$ is the solvent density. It then follows that the two second virial coefficients are related by the expression (Wills et al. 1993)

12 $$C_{22} \, = \,\left( {B_{22} - M_{2}. \overline{v}_{2} } \right)\rho_{1}$$

which allows conversion of the molal second virial coefficient ${C}_{22}$ to its molar osmotic second virial counterpart ${B}_{22}$_._

The above consideration of nonideality under the constraints of fixed temperature and pressure does, of course, refer to a single-solute system—a protein dissolved in solvent (water). As noted in the early days of static light scattering measurements (Kirkwood and Goldberg 1950; Stockmayer 1950) the monitoring of solute thermodynamic nonideality on the molal scale mandates the inclusion of additional virial coefficient terms for protein interaction with buffer components and other small cosolutes, which cannot be regarded as part of the solvent. It therefore becomes necessary to distinguish between nonideality reflecting the second virial coefficient for protein self-interaction (${C}_{22}$) and that emanating from physical protein–cosolute interaction (designated for simplicity of presentation as ${C}_{23}$). The previous expression for the activity coefficient [Eq. (3)] needs modification to the form

13 $${\text{ln}}\,{\text{y}}_{2} \, = \,2C_{22}. m_{2} + C_{23}. \,m_{3} + ...$$

to account for the physical interaction between protein and the cosolute present at molal concentration ${m}_{3}$.

More than fifty years elapsed before these requirements resurfaced in an investigation (Winzor et al. 2007) designed to test whether nondeality arising from protein–cosolute interactions could account for the negative light scattering second virial coefficients obtained in the presence of high concentrations of uncharged cosolutes, such as polyethylene glycol (Vivarés and Bonneté 2002) and sucrose (Winzor et al. (2007), for which nonideality reflects only the hard-sphere excluded volume interactions. Interest in this possibility was triggered by the presence of the term–$\left( {C_{23} /\rho_{1} } \right)^{2}$ in the expression for the light scattering second virial coefficient (Kirkwood and Goldberg 1950; Winzor et al. 2007). Although that endeavour did lead to a decrease in the magnitude of ${A}_{2}$, the effect was minor compared to the experimentally observed dependence of ${A}_{2}$ upon ${C}_{3.}$ Furthermore, those calculations neglected the contribution of a third virial coefficient term, $C_{223} /\rho_{2}^{2}$, which, it transpires, effectively counters that from$\left( {C_{23} /\rho_{1} } \right)^{2}$. *C*_223_ is a parameter with the dimensions of a third virial coefficient reflecting the potential-of-mean-force interaction of a single cosolute molecule, which may be an electrolyte with a pair of protein molecules, and about which little is known, so it is effectively a fitting parameter (Deszczynski et al. 2006).

For present purposes we therefore proceed on the basis that the effects of thermodynamic nonideality in light scattering measurements on buffered aqueous protein solutions can be described adequately by single-solute theory (the standard practice). The respective expressions for *m*_2_ and *C*_22_ continue to be given by Eqs. 11 and 12, but with ρ_1 _the density of the cosolute-supplemented solvent.

Because the thermodynamic excluded volume parameter can only assume positive values, we also examine the possibility that the negative values of ${A}_{2}$ reported in the literature may include hydrodynamic contributions.

## Macromolecular solutions: a hydrodynamic perspective

The first detailed consideration of the effect of hydrodynamic interactions on Brownian motion involving the net flux of solute was provided by Batchelor (1976) in the context of concentration-dependent diffusion in sedimentation velocity for solutions dilute enough that only pairwise interactions between particles were significant. In that study a combination of statistical-mechanical and hydrodynamic approaches led to description of the concentration dependence of the diffusion coefficient *D* for a rigid, uncharged spherical particle under such very dilute conditions as

14 $$D\,\, = \,\,\,\frac{{\left( {{\text{k}}_{{\text{B}}} T} \right)}}{{6\pi \eta_{1} R_{2} }}\left[ {1 + \left( {8\phi - 6.55\phi } \right)} \right]\, = \,D_{o} \left[ {1 + \left( {{\uplambda }_{T} - \lambda_{H} } \right)\phi } \right] = D_{o} \left( {1 + k_{D}. c_{2} } \right)$$

in which $\phi$, the volume fraction occupied by the diffusing particle = *c*_2_.*v*_s_, the product of the weight-concentration ${c}_{2}$ (g/ml) of the solute and its solvated specific volume (ml/g)${\nu}_{s} = 4\pi N_{A} R_{2}^{3} /\left( {3M_{2} } \right)$ . $\lambda_{T}$ and $\lambda_{H}$ are coefficients representing the thermodynamic and hydrodynamic nonideality coefficients (the latter equivalent to *K*_s_ in Harding and Johnson (1985a, b)). $D_{o} = \left( {{\text{k}}_{{\text{B}}} T} \right)/(6\pi \eta_{1} R_{2})$ is the “ideal” translational diffusion coefficient obtained experimentally in the limit of zero solute concentration. From Eq. (7) it is evident that the factor ${\lambda }_{T}=$ 8 in Eq. (14) corresponds to $2{B}_{22}^{HS}/{M}_{2}$, the volume from which the centres of two uncharged solute molecules are mutually excluded; and is therefore a thermodynamic factor. Hydrodynamic factors are incorporated into the second term, ${\lambda }_{H}=$ 6.55 of Eq. (14), which decreases the effective magnitude of the excluded volume. It is worth noting that for more concentrated solutions the pairwise approximation ceases to become valid: from multi-particle theory Brady and Durlofsky (1988) obtained a value of 5 for hard spheres, also from a solvent frame of reference (Harding and Johnson 1985a, b).

On the grounds that the osmotic second virial coefficient equates with half of the thermodynamic contribution to excluded volume, the same situation also applies to its hydrodynamic counterpart. The excluded volume contribution to an experimentally measured concentration coefficient ${k}_{D}$ (mL/g) in Eq. (14) thus becomes [$\left({\lambda }_{T}-{\lambda }_{H}\right){v}_{s}]$/2 to be consistent with the description of ${\lambda }_{T}$ as $2{B}_{22}^{HS}/{M}_{2}$.

Most of the subsequent attention has been directed towards the determination of *D* from dynamic light scattering studies, for which the same expression [Eq. (14)] has also been obtained for an uncharged spherical particle (Felderhof (1978; Wills 1979; Phillies and Wills 1981; Cichocki and Felderhof 1988). Petsev and Denkov (1992) have shown that the presence of net charge on those hard spheres increases the magnitude of the thermodynamic term in accordance with Eqs. (4a,4b) and (5). The corresponding relationship for the hydrodynamic term is dominated by the Oseen contribution)$\left( {\lambda_{o} v_{s} } \right)$

15 $$\lambda_{o} v_{s} = \frac{{2\pi N_{A} R_{2} }}{{M_{2} }}\left[ {\int_{0}^{{2R_{2} }} {rdr + \int_{2{R_{2}}}^{\infty } {f_{22} rdr} } } \right]$$

which establishes that the Oseen hard-sphere contribution to ${\lambda }_{H}$ is 6: the remainder (0.55) comes from two other contributions that account for short-range hydrodynamic interactions (Felderhof 1978; Petsev et al. 1992).

The coefficient describing the concentration dependence of the translational diffusion coefficient, ${k}_{D}$(mL/g) = (${\lambda }_{T}-{\lambda }_{H}){v}_{s}/2$, for charged hard spheres (HS) has therefore been considered to be given by the expression

16 $$k_{D} = \frac{{\left( {\lambda_{T}^{HS} - \lambda_{H}^{HS} } \right)v_{s} }}{2} + \frac{{2\pi{\text{N}}_{{\text{A}}} }}{{M_{2} }}\mathop \int \limits_{{2R_{2} }}^{\infty } f_{22} \left( r \right) r^{2} dr + \frac{{2\pi N_{A} R_{2} }}{{M_{2} }}\mathop \int \limits_{{2R_{2} }}^{\infty } f_{22} \left( r \right)rdr$$

with ${f}_{22}\left(r\right)$ defined by Eqs. (4b) and (5). Although the two integrals in Eq. (16) are usually evaluated by expanding the exponential in ${f}_{22}(r)$ as a power series in *r*, the value obtained for the electrostatic contribution to the excluded volume (${\lambda }_{T}{v}_{s}$) is an overestimate (Wills and Winzor 2009); and a similar situation presumably applies to the corresponding contribution to the hydrodynamic term ${\lambda }_{O}{v}_{s}$ in Eq. (15). We have therefore used the trapezoidal integration procedure for estimates of $({\lambda }_{T}-{\lambda }_{H}){v}_{s}$ in considerations of experimental systems

In dynamic light scattering studies $({\lambda }_{T}^{HS}-{\lambda }_{H}^{HS}$) has often been taken as 1.45 for dilute solutions of rigid spherical particles in dominant Brownian motion, the value deduced by Batchelor (1976) for sedimentation velocity and traditional diffusion measurements, where particle flux is effected by a concentration gradient. It is worth pointing out that this value however only applies to *very* dilute conditions where the pairwise approximation is valid (Brady and Durlofsky 1988; Winzor et al 2021). Use of this value in dynamic light scattering studies has also been criticized by Phillies (1987) on the grounds that light scattering spectroscopy is merely sensitive to particle position; and that the value of $({\lambda }_{T}^{HS}-{\lambda }_{H}^{HS}$) should therefore be decreased to − 0.9.

## Analysis of experimental results

**Fig. 1:**
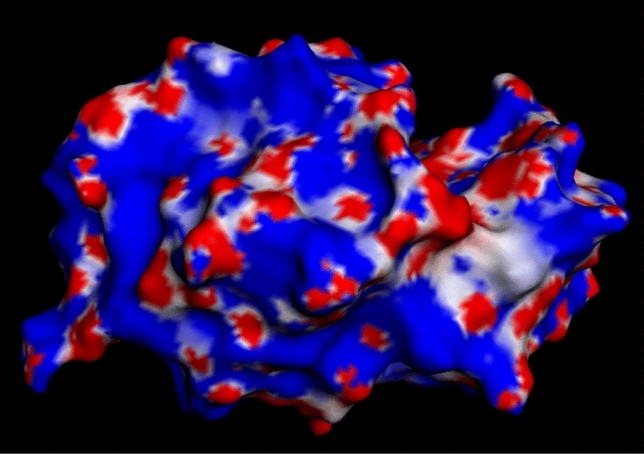
Electrostatic map calculated using the Poisson–Boltzmann equation of hen egg white lysozyme (PDB: 1AKI) at pH 7.0. It can be clearly seen that there is an even distribution of charge across the surface (positive: blue; negative: red). Hydrophobic patches are shown in grey: the only patch visible was c.a. 5 angstroms in diameter seen in the bottom right of the molecule, and is unlikely to contribute to aggregation due to the large electrostatic shadow cast by the other residues in the molecule. No other patches were visible on the molecule (data not shown) (color figure online)

**Fig. 2:**
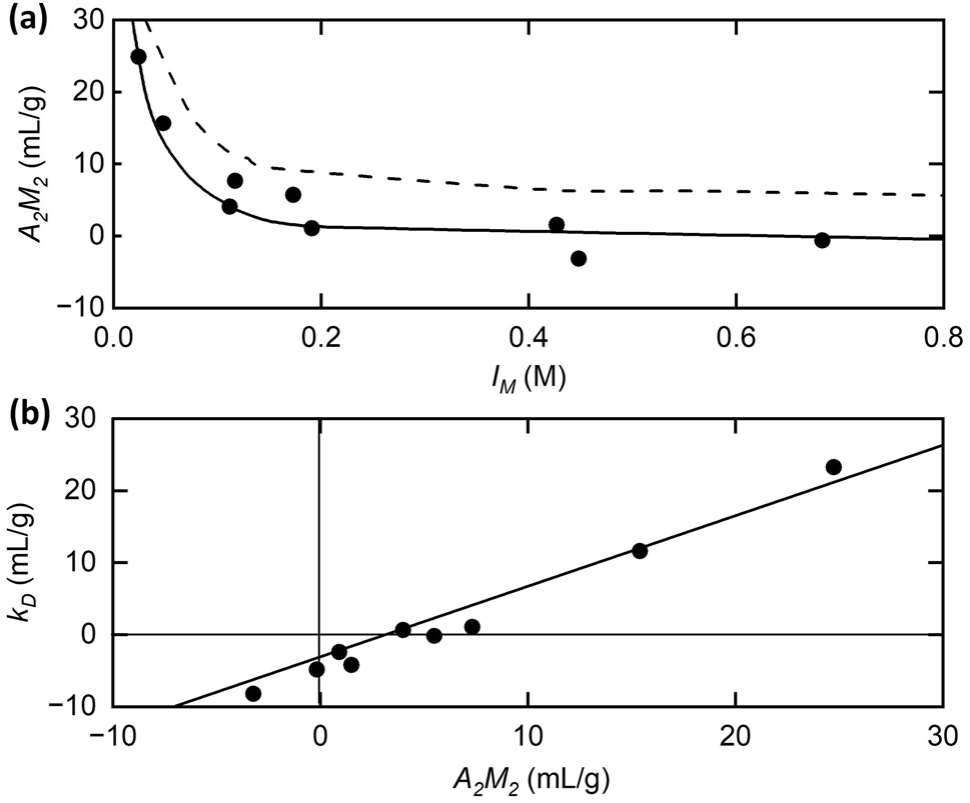
Analysis of static light scattering data for lysozyme solutions at pH 4.7 (from data of Muschol and Rosenberger 1995). **a** Experimental results $\left( \bullet \right)$ for the dependence of the second virial coefficient ${A}_{2}{M}_{2}$ upon ionic strength, together with theoretical dependence predicted either on the basis of its consideration as the equivalent of the osmotic second virial coefficient for protein self-interaction, ${\lambda }_{T}{v}_{s}/2$ $\left( { - - - } \right)$, or the combination of that parameter and its hydrodynamic counterpart (—), as in Eq. (16) for the diffusion concentration dependence coefficient, $\left({\lambda }_{T}-{\lambda }_{H}\right){v}_{s}/2$. **b** Demonstration of the correlation between ${k}_{D}$ and ${A}_{2}{M}_{2}$. [Data for ${\lambda }_{T}$ and ${\lambda }_{H}$ calculated from Table 2 of Muschol and Rosenberger (1995)]

Lysozyme represents a good starting point for analysis, due to its low degree of asymmetry (Blake et al 1965) and approximate uniform surface charge distribution (Fig. 1). The ionic strength dependence of the light scattering second virial coefficient for lysozyme in acetate and acetate–chloride buffers (pH 4.7) is presented in Fig. 2a, where the experimental points $\left( \bullet \right)$ have been calculated from the values of ${A}_{2}$ reported in Table 2 of Muschol and Rosenberger (1995) and a molecular mass of 14,600 Da. Attempts to describe these data in thermodynamic terms, ${2A}_{2}{M}_{2}={\lambda }_{T}{v}_{s}-{\overline{v} }_{2}$ $,$ with respective values of 1.9 nm and 11 for ${R}_{2}$ and ${Z}_{2}$ (Muschol and Rosenberger 1995) to solve the integrals in Eq. (4a,b) lead to their consistent overestimation $(- - -)$.

This finding clearly corroborates earlier assertions that the light scattering coefficient should not be regarded as the osmotic second virial coefficient (Deszczynski et al 2006; Winzor et al. 2007; Wills et al. 2015). Much better agreement between experiment and prediction is achieved (^**_______**^) by adopting the viewpoint that $2{A}_{2}{M}_{2}$ monitors [(${\lambda }_{T}-{\lambda }_{H}){v}_{s}-{\overline{v} }_{2}]$ with the additional hydrodynamic term in $\lambda_{H}$ (see also Harding and Johnson 1985a, b), an observation that favours consideration of the light scattering coefficient as a hydrodynamic rather than an equilibrium parameter. From Eq. (16) it is evident that the concentration dependence of ${A}_{2}{M}_{2}$ should then parallel that for the diffusion coefficient, which also monitors [$({\lambda }_{T}-{\lambda }_{H}){v}_{s}/2]-{\overline{v} }_{2}$. The small extra term ${\overline{v} }_{2}$ comes from the Gibbs–Duhem relation (Harding & Johnson 1985a).

The extent of that correlation is shown in Fig. 2b, where the values of $${k}_{D}$$ have also been calculated from Table 2 of Muschol and Rosenberger (1995) after correcting for their use of the unsolvated mole fraction ($${\overline{v} }_{2})$$ for $${v}_{s}$$. Although those results conform reasonably well with the concept of a linear relationship between $${k}_{D}$$ and $${A}_{2}{M}_{2} $$ with a slope of unity, the observation of a finite (negative) ordinate intercept precludes their consideration as the same parameter.

Inasmuch as Eq. (16) implies constancy of viscosity (taken as that of buffer, $\eta_{b}$ ), no account has been taken of the effect of solution viscosity on the magnitude of the measured diffusion coefficient at finite protein concentrations. This deficiency is remedied by writing the concentration dependence of the diffusion coefficient as (Scott et al. 2014)

17 $$D = D_{o} \,\frac{{1 + k_{D} .c_{2} }}{{\eta /\eta_{b} }}$$

where η denotes the viscosity of a protein solution with concentration ${c}_{2}$ for which *D* was measured, and $D_{o}$ that of buffer (the solution viscosity in the limit of zero solute concentration to which ${D}_{o}$ refers). Allowance for the fact that the relative viscosity is related to the intrinsic viscosity $\left[ \eta \right]$ of a spherical protein species by the expression (Tanford 1961; Harding 1997)

18 $$\eta /\eta_{b} = 1 + \left[ \eta \right].c_{2 + \ldots } \approx 1 + 2.5v_{s} c_{2}$$

introduces an additional decrease in the predicted value of ${k}_{D}$ by 2.5 ${v}_{S}$. An ordinate intercept of − 2.5 ${v}_{s}$ and a slope of unity is thus the predicted dependence of ${k}_{D}$ upon ${A}_{2}{M}_{2}$ (— in Fig. 2b). This is close to the intercept value of ~ − 3.1 ${v}_{s}.$ Exact agreement is made if allowance is made for the strong dependence of the Einstein-Simha viscosity shape factor ν on shape (ν = 2.5 for spheres and > 2.5 for other shapes), and can be determined exactly from triaxial crystallographic dimensions (Harding 1982, 1997; Harding et al 1979, 1981, 1982, 1983, 2005). From its crystallographic dimensions (Blake et al 1965) lysozyme approximates a prolate ellipsoid of axial ratio ~ 2.1. Using the program ELLIPS1 (Harding et al 2005; Garcia de la Torre & Harding 2013) this corresponds to a value of ν = 3.1. By contrast the exclusion volume term *A*_2_*M*_2_ is relatively insensitive to such a shape change (Rallison & Harding 1985; Harding et al 1999). The degree of conformity between experiment and prediction is considered excellent for the reported data even with no indication of experimental uncertainty inherent in the measurements.

**Fig. 3:**
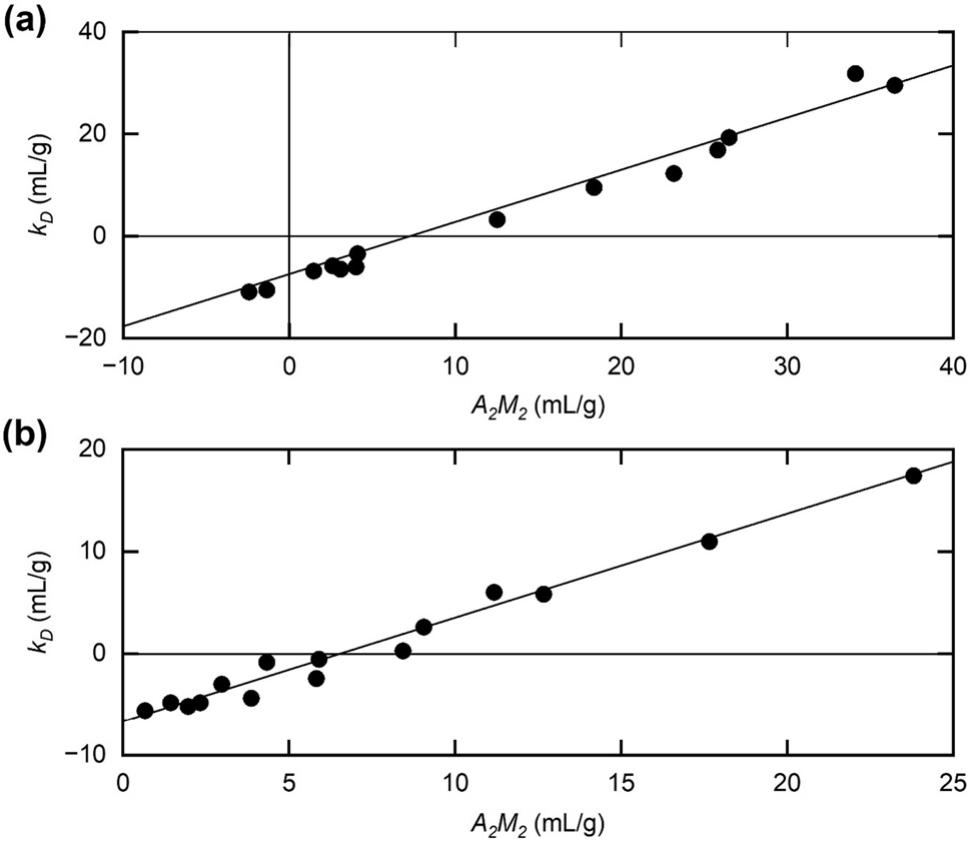
Further evidence for correlation between the diffusion concentration dependence coefficient ${k}_{D}$ and the light scattering second virial coefficient ${A}_{2}{M}_{2}$ for monoclonal IgG antibodies. **a** Combined results for five monoclonal IgG antibodies (pH 6.0) at high and low ionic strengths. [Data taken from Lehermayr et al (2011)] **b** Corresponding dependence for a single monoclonal antibody at pH 5.0 and pH 5.75 and a range of ionic strengths. [Data from Roberts et al. (2014)]

Similar linear plots for the dependence of $k_{D}$ upon $A_{2} M_{2}$ have been reported for five monoclonal antibodies in histidine–chloride buffers (pH 6.0) with low and high ionic strengths (Lehermayr et al. 2011), and also for a single monoclonal antibody over a range of pH and ionic strengths (Roberts et al. 2014). Data from the former study are shown in Fig. 3a, and those at pH 5.0 and pH 5.75 from the latter investigation in Fig. 3b. A molar mass ${M}_{2}$ of 145 kDa and a Stokes radius ${R}_{2}$ of 5.2 nm (Roberts et al. 2014) have been used to calculate the predicted ordinate intercepts. As in Fig. 2b, the experimental results conform reasonably well with a slope of unity (— in Fig. 3a, b), although the intercepts are ~ 7 mL/g, considerably differ from the predicted − 2.5 ${v}_{S}$ for hard spheres. However, again if we allow for the greater sensitivity to shape of the viscosity increment ν compared with the exclusion volume: the value for the Einstein-Simha viscosity increment ν for an IgG antibody of  molar mass ~ 150,000 g/mol is ~ 4.3 (Longman et al 2005). The time averaged hydration δ for antibodies is ~ 0.6 g water/g protein (Lu et al 2006) so ${v}_{S}$ = (${\overline{v} }_{2}$+ δ/ρ_o_) ~ 1.35 mL/g, where ρ_o_ is the density of the aqueous solvent (~ 1 g/L). So the predicted value for the intercept = − ν$.{v}_{S}$ is ~ 6–7 mL/g and appears to be consistent with Fig. 3a and b, and the experimentally measured values for the intrinsic viscosity (Kilar et al. 1985; Longman et al 2005).

## Concluding remarks

The ionic strength dependence of ${A}_{2}{M}_{2}$ for lysozyme (pH 4.7) shown in Fig. 2a has again challenged the designation of this light scattering coefficient as the osmotic second virial coefficient, either in its molar form or its molal form ${C}_{22}{.M}_{2}={\lambda }_{T}{v}_{s}/2-{\overline{v} }_{2}$, which is unequivocally a thermodynamic parameter. Further support for the need to consider the Rayleigh ratio $R_{\theta }$ obtained from “static” light scattering intensity as a steady-state rather than an equilibrium measurement comes from Figs. 2b, 3a b, which provide direct experimental evidence of a correlation between ${A}_{2}{M}_{2}$ and the corresponding parameter, ${k}_{D}$, for concentration dependence of the translational diffusion coefficient. This reflects the difference between the consequences of excluded volume and hydrodynamic intermolecular interactions. In other words, evidence is mounting for identification of the light scattering second virial coefficient as (${\lambda }_{T}-{\lambda }_{H}){v}_{s}/2$ rather than ${\lambda }_{T}{v}_{s}/2$, and hence for cessation of its consideration as a thermodynamic parameter.

By demonstrating that the light scattering second virial coefficient ${A}_{2}{M}_{2}$ equates with the excluded volume contribution to ${k}_{D}$, the coefficient describing the concentraton dependence of diffusion coefficients obtained by dynamic light scattering, this investigation has substantiated our earlier conclusion (Deszczynski et al. 2006; Winzor et al. 2007) that ${A}_{2}$ should not be designated as the osmotic second virial coefficient for protein self-interaction, $B_{22} /M_{2}^{2}$ – a parameter with thermodynamic status (McMillan and Mayer 1945). A subsequent challenge to that conclusion (Blanco et al. 2011) perhaps reflected an entrenched historical practice whereby the nonideality parameter emanating from scattering spectroscopy measurements (visible light, X-rays, neutrons) is automatically described as the osmotic second virial coefficient. Indeed, the possibility that negative values of ${A}_{2}$ might include some consequences of hydrodynamic interactions had already been suggested (Neal et al. 1999). That suggestion appears to be further supported by the demonstration that ${A}_{2}{M}_{2}$ monitors (${\lambda }_{T}-{\lambda }_{H}$), the difference between thermodynamic and hydrodynamic excluded volume contributions, respectively; and thereby appears to invalidate the implication inherent in the light scattering literature that ${A}_{2}$ is the thermodynamic parameter ${B}_{2}$ (= *B*_22_*/M* ^2^) in mL.mol/g^2^. Specifically

19 $$A_{2} M_{2} = B_{2} M_{2} - \overline{v}_{2} - \lambda_{H} v_{s} /2$$

A second problem addressed in this investigation has been the failure of the dependence of ${k}_{D}$ upon ${A}_{2}{M}_{2}$ to pass through the origin despite the 1:1 correlation. That dilemma has been overcome by incorporating the additional effect of solution viscosity on D–c dependence, a factor neglected by Batchelor (1976) and Felderhof (1978) in the rationalization of nonideality on the statistical-mechanical basis of excluded volume.

Finally, some consideration needs to be given to the consequences of the current reclassification of ${A}_{2}$ as a non-thermodynamic parameter on recent procedures for the quantitative characterization of nonideality in static light scattering measurements (Minton 2007; Fernàndez and Minton 2009). From the theoretical expression that forms the basis of the analysis for a single uncharged solute,

20 $$\frac{{R_{\theta } }}{K} = \,\frac{{M_{2} c_{2} }}{{1 + c_{2} \,d\,{\text{l}}{\text{n}} \,y_{2} /dc_{2} }} = \frac{{M_{2} c_{2} }}{{1 + 8v_{s} c_{2} }}$$

it is evident that Rayleigh scattering ratio $R_{\theta }$ is being accorded full thermodynamic status: $8{v}_{s}$ is $\lambda_{T}$ for an uncharged sphere in the above considerations. Incorporation of the increased excluded volume for a charged species, $\lambda_{T}^{EL} ,$ is accommodated (Minton and Edelhoch 1982) by increasing the effective size of the hard sphere by expressing Eq. (7) as

21 $$B_{22} = \frac{{16\pi {\text{N}}_{{\text{A}}} (R_{eff}^{HS} )^{3} }}{3}$$

The method is empirical in the sense that a value for the solvated specific volume is obtained as a curve-fiting parameter, whereupon the contributions of the hydrodynamic terms $\lambda_{H}^{HS} ,\,\lambda_{H}^{EL}$ are also incorporated. It therefore remains valid as an empirical procedure despite being based on thermodynamic expressions that do not apply to static light scattering measurements. There is also potential for error in the analysis arising from the other curve-fitting parameter, which has been taken as ${M}_{2}$ in Eq. (19) but which incorporates a protein–cosolute virial coefficient term ${B}_{23}{C}_{3}$ because of the need to regard buffer species as additional non-scattering cosolutes (Kirkwood and Goldberg 1950; Winzor et al. 2007; Blanco et al. 2011; Wills and Winzor 2017).

The combination of empiricism and the non-equilibrium nature of $A_{2}$ clearly detracts from “static” light scattering as an alternative to techniques such as osmometry and sedimentation equilibrium, where conformity with the constraints of constant temperature and solvent chemical potential fully justify the interpretation of experimental data in terms of thermodynamic expressions for single-solute systems. This can also have a bearing on the analysis of reversible self-association processes. With sedimentation equilibrium (and osmotic pressure the effects of reversible self-association are nonetheless difficult to extract from thermodynamic nonideality effects as they are both dependent on concentration (see Scott and Winzor 2009 and Harding and Rowe 2010) and sometimes these effects can cancel out to lead to “pseudo-ideal” conditions. Where structural information about the monomer species is available, for globular based structures the best procedure is to calculate *B*_2_ or *B*_22_ from the tri-axial shape of the molecule (and the net charge and ionic strength) using a routine COVOL, downloadable from https://www.nottingham.ac.uk/ncmh/ (Harding et al 1998, 1999; Harding 2013)—or for asymmetric shapes such as immunoglobulins using SOLPRO (Garcia de la Torre et al. 1999). With light scattering there may be an extra layer of difficulty with having the added complication of hydrodynamic as well as thermodynamic nonideality to deal with.
